# Tibia Fractures in Children: A Single-Centre 11-Year Retrospective Study for Evaluating the Management and Outcomes in an Acute General Orthopaedic Hospital

**DOI:** 10.7759/cureus.36462

**Published:** 2023-03-21

**Authors:** Shubhendu Chakraborty, Karim Salama, Ling Hong Lee

**Affiliations:** 1 Trauma and Orthopaedics, Salisbury District Hospital, Salisbury, GBR; 2 Trauma and Orthopaedics, Sunderland Royal Hospital, Sunderland, GBR

**Keywords:** outcomes, managements in theatre, displaced fractures, children, tibia fractures

## Abstract

Introduction

Tibia fractures are children's most typical lower limb fractures affecting their general and mental well-being. This study aims to evaluate the management and outcomes of displaced tibia fractures.

Methods

A retrospective study was conducted to review children up to 16 years of age with displaced tibia shaft fractures who received treatment in our department from January 2011 to December 2021. Fractures managed in the operating theatre and patients who completed follow-up until fracture healing were included in the study. Treatment procedures were assessed, and outcome was measured by hospital stays, complications and revision procedure incidences.

Results

The study included 74 patients (75 fractures, including one re-fracture). The patient's ages ranged from 2 to 16 years (median age: 11 years). Seven patients sustained open fractures (Gustilo I, II). Tibia diaphysis was the most common site of involvement. A total of 43 patients were treated by manipulation under anaesthesia and cast. Surgical fixation was directly proportional to increasing age (p<0.05). Overall, 74% of patients were treated by fixation when age was >10 years. Three patients needed conversion of casting to surgical fixation. One patient had re-fracture following a secondary injury after six months of initial tibia shaft fracture treated by casting. Five patients had complications, including delayed union, pin site and surgical site infections. Antibiotics were adequate to manage infections except in one patient who needed debridement. The average hospital stay was three days. The median number of follow-up X-rays was 4. The planned removal of all flexible nails, and the circular frame was done between 2 and 15 months, except for one that had delayed union. All the patients underwent clinical and radiological union at the end of the follow-up.

Conclusion

The treatment plan was dependent on the individual need of the patient and the fracture pattern. Children older than 10 years were more likely to undergo surgical fixation. The majority of fractures were treated by manipulation and cast in operating theatres. Better logistic support in the emergency department could reduce the burden on the operating theatre.

## Introduction

Children are at a higher risk of fractures than other age groups, especially fractures of the long bone, which significantly affects their daily living, health and well-being [1,2]. The tibia fracture is the most common fracture following upper limb fractures [3], accounting for around one-fifth of paediatric fracture-related hospital admissions [4]. Thus this is a vital public health concern [1].

There is a range of management strategies, including conservative and surgical options, for tibia fractures in children [5,6]. A significant proportion of such fractures can be treated by plaster casting due to the presence of thick periosteum, good blood supply and remodelling potential, allowing for satisfactory and quick fracture union. However, surgical management is sometimes appropriate based on patient characteristics and fracture patterns [7]. Improvement and innovation in surgical techniques, a drive to reduce hospital stays and early mobilization for rehabilitation are also factors influencing surgical decisions [8,9].

This study's objective is primarily to evaluate the management of displaced tibia shaft and metaphyseal fractures in children. Secondly, we aim to assess our treatment outcomes for these fractures.

## Materials and methods

This was a single-centre retrospective analysis conducted out in a district general hospital having trauma and orthopaedic department in the United Kingdom.

Patients were identified retrospectively using a local perspective trauma database. Inclusion criteria were patients up to 16 years old with displaced tibia shaft and metaphyseal fractures who were taken to the operating theatre for definitive management between January 1, 2011, and December 31, 2021. This study was registered with the local audit department as a service evaluation.

Fractures of the proximal or distal growth plate or epiphysis, tibia spine or tibia tuberosity were excluded. Patients who had their fractures managed definitely in the outpatient settings or who could not meet the minimum follow-up criteria or were transferred to other hospitals were also not included in the study. Clinical records were obtained to determine the demographics, associated injuries, closed or open fractures, management methods, perioperative issues, clinical healing and patient outcomes. Outcome measures were evaluated in terms of length of hospital stay, post-operative complications and re-operation incidence.

Multiple consultants managed patients on the trauma rota, including two with paediatric orthopaedic fellowship experience. Indications for management in the operating theatre were open fractures, displaced or unstable fractures not successfully reduced or maintained in plaster of Paris/cast applied in the emergency department. The definitive treatment used was at the discretion of the treating surgeon, with the involvement of the parents and the injured child.

Patients were mobilized as per the post-procedure instructions, which included full weight-bearing mobilization as tolerated on the first post-operative day, non-weight-bearing mobilization for four to six weeks or more and staged weight-bearing status as per the need of the individual patient. All the patients were followed up until clinical or radiological union.

The study did not receive any funding to conduct.

## Results

Between January 2011 and December 2021, 74 patients under 16 years of age (75 tibia fractures and one re-fracture), who fulfilled the inclusion criteria were treated in our department. The age range was 2 to 16 years, with a median age of 11 years. The male (n=50) to female (n=24) ratio was 2.1:1. In addition, seven patients sustained open fractures (six had Gustilo-Anderson type I and one had Gustilo-Anderson type II fracture).

**Figure 1:**
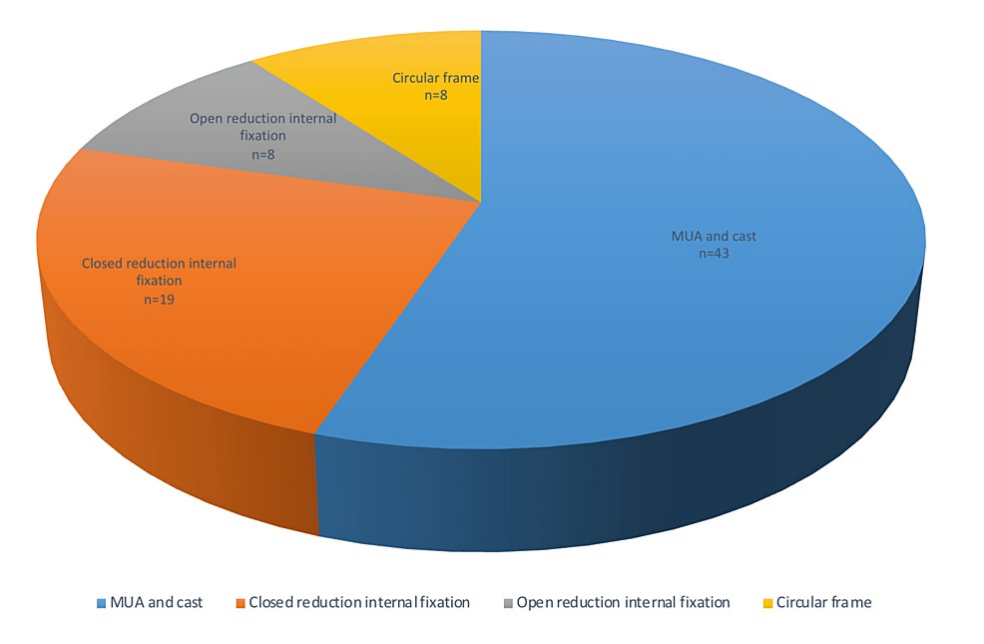
Types of management.

**Figure 2:**
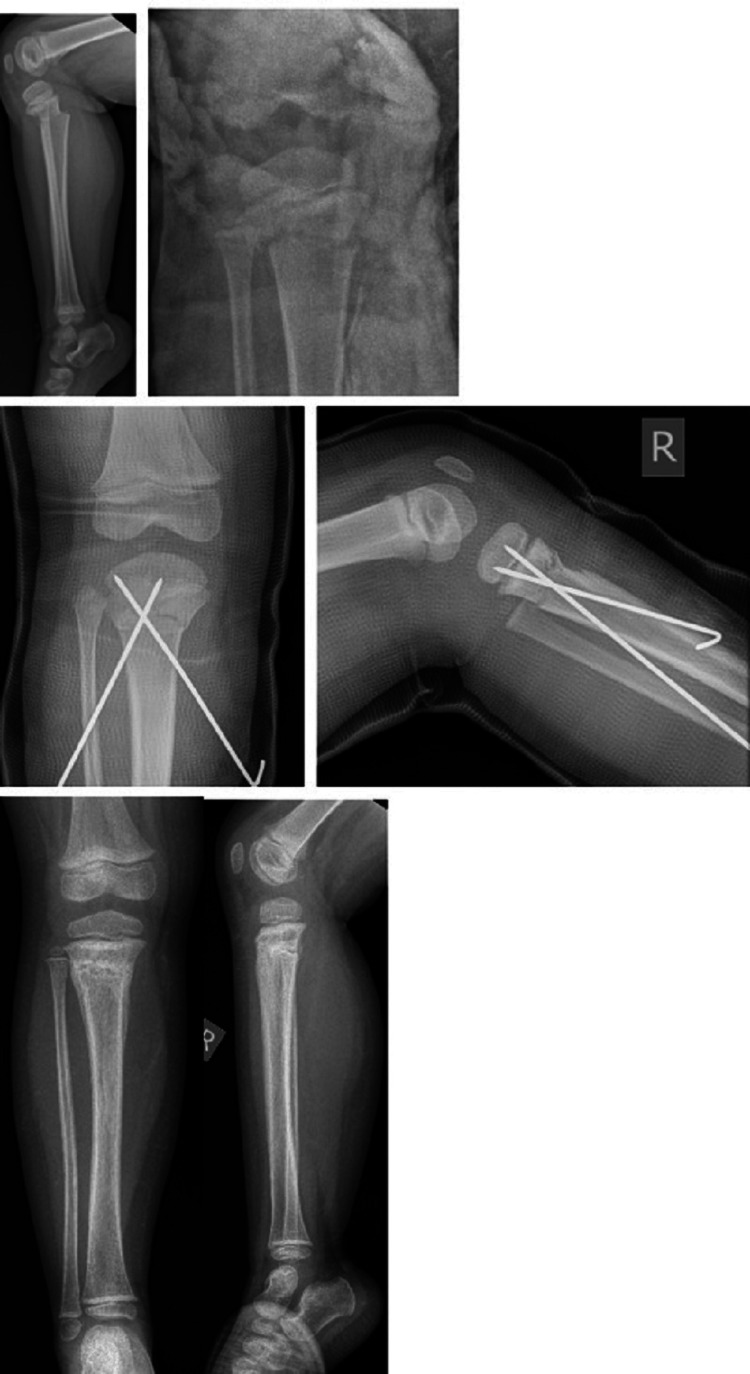
A 3-year-old girl with proximal tibia fracture. Closed reduction and internal fixation was performed with percutaneous retrograde 2mm Kirschner wires. Wires were removed five weeks post-operatively.

**Figure 3:**
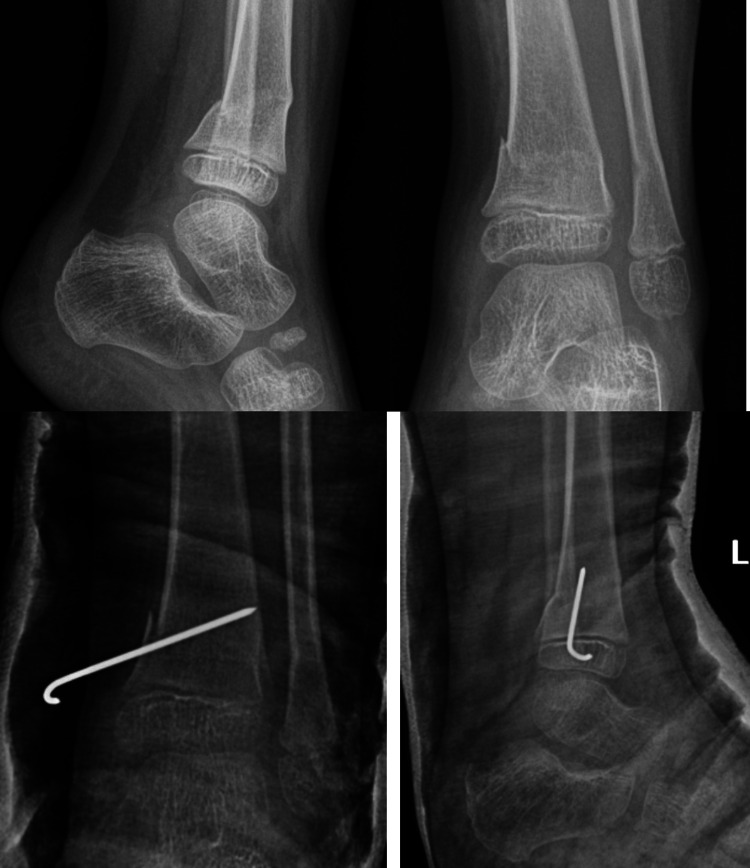
A 6-year-old boy with distal metaphyseal fracture. Closed reduction and fixation was performed with Kirschner wires. Wire was removed five weeks post-operatively.

**Figure 4:**
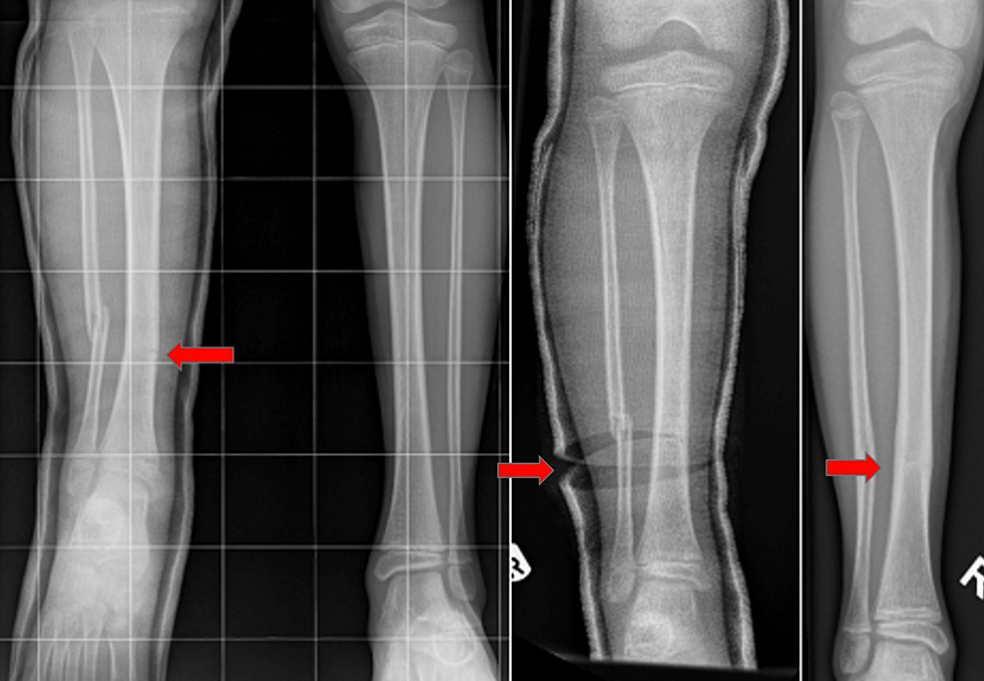
An 11-year-old boy with fracture of tibia diaphysis and fibula at the same level. From left to right: fracture angulated at two weeks’ review following MUA in theatre (radiographs in left), wedge created in the casts (radiograph in the middle), and satisfactory healing and alignment four months after initial casting (radiograph in the right). Arrow in the first radiograph shows angulation at the fracture site in the anteroposterior view. Arrow in the third picture shows wedging of the cast resulting in correction of angulation. Arrow in the fourth picture shows satisfactory healing with acceptable angulation at the fracture site. MUA, manipulation under anaesthesia

**Figure 5:**
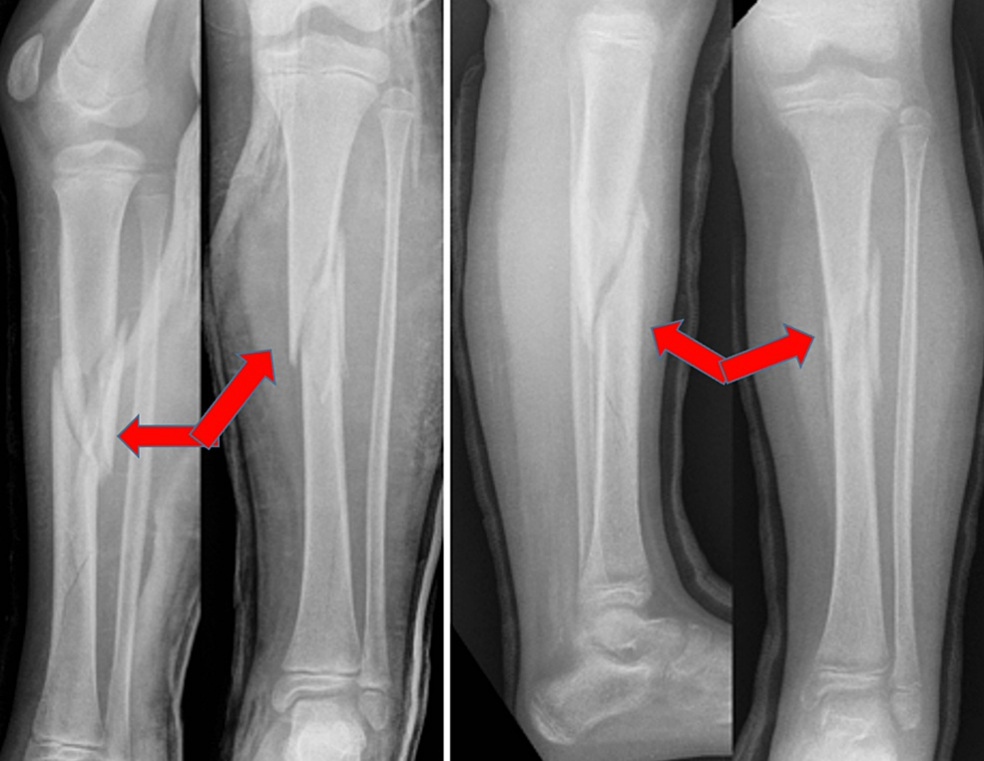
A 9-year-old girl with multi-fragmented displaced fracture (six weeks after manipulation and casting). Arrows in the first and second images show multi-fragmentary fracture. Third and fourth images show satisfactory healing in the anteroposterior and lateral views.

Tibia diaphysis was the most frequent (n=39, 53%) site of involvement (Table 1). More than half (n=43, 55%) of the patients were managed by manipulation under anaesthesia (MUA) and plaster/casting. The remaining 31 patients underwent surgical fixation (Figure 1). The incidence of surgical fixation was directly proportional to increasing age (p<0.05); 89% of the children aged five years or younger were managed conservatively, compared to 74% who needed surgical fixation of the fracture when the age was 10 or more. Table 2 shows a schematic presentation of different fixation methods. Closed reduction internal fixation (CRIF) (n=19) was performed by employing flexible nailing (n=14), Kirschner wire (K-wire) fixations (n=2) (Figures 2, 3) and intramedullary (IM) nailing (n=3, in skeletally mature patients or whose physis was closed). Open reduction internal fixation (ORIF) was performed by flexible nailing (n=1), screw fixation (n=1) and plate fixations (n=6). Table 3 shows the different types of plates used according to the location of the fracture site. Plates were contoured to fit bone surface or for compression as needed by the operating surgeon. The circular frame using the Taylor spatial frame (TSF) system was used as the external fixation method. Table 1 also describes a management method according to the involvement sites. Among the patients who underwent MUA/cast, two underwent successful wedging to improve angulation (Figure 4), and two fractures were multi-fragmentary (Figure 5).

**Table 1:** Tibial fracture sites of involvement along with management according to site. One re-fracture of diaphyseal fracture happened, which makes n=40 IM nail, intramedullary nail; K-wires, Kirschner wires; MUA, manipulation under anaesthesia

Site	Frequency	Percent	Management
Proximal metaphysis	1	1	K-wire 1
Meta-diaphysis	3	4	MUA 2, plate 1
Diaphysis	39	53	Flexible nail 13, IM nail 1, plate 3, circular frame 4, MUA 19
Dia-metaphysis	15	20	Screw 1, flexible nail 1, IM nail 2, plate 1, circular frame 3, MUA 7
Distal metaphysis	16	22	Flexible nail 1, plate 1, k-wire 1, circular frame 1, MUA 12
Total	74	100.0	

**Table 2:** Different methods of fixation/management. Two MUA converted into further fixation, therefore, n=41 instead of 43 in MUA IM nail, intramedullary nail; K-wires, Kirschner wires; MUA, manipulation under anaesthesia

Implant/management type	Frequency	Percent
MUA and cast	41	54
Screw	1	1
Flexible nail	15	20
IM nail	3	4
K-wires	2	3
Plate	6	8
Circular frame	8	10
Total	76	100

**Table 3:** Types of plates used according to fracture location. LCP, locking compression plate

Fracture location	Plate used
Distal dia-metaphyseal junction	3.5mm LCP straight plate
Diaphyseal (x3)	3.5mm LCP straight plate 4.5mm narrow LCP straight plate (x2)
Proximal meta-diaphyseal junction, recent tibia tubercle osteotomy	4.5mm proximal lateral tibia LCP plate
Distal metaphyseal	3.5mm T-plate for tibia, 2.4mm LCP straight plate fibula

 

One patient had screws-only fixation. This patient had a concurrent infection of an ingrown toenail on the same side of the distal tibia spiral fracture. Minimal metalware as needed (two compression screws) was chosen to reduce the risk of deep tissue infection. The fracture was not stable enough to be immobilized in plaster only. We could not accurately review the exact indication and post-operative mobilization following other fixation methods due to the retrospective nature of this study. But, in general, fixation was used for fracture patterns considered unstable and unsuitable for a plaster cast. The choice of the implant was dependent on the operating surgeon’s experience, the skeletal maturity of the patient, the fracture pattern and the patient’s age. Circular frame, in general, was chosen because of the difficult location/configuration of the fracture. In addition, a circular frame was applied in open fracture, where there was soft tissue concern, to avoid internal fixation or to allow earlier mobilization.

Three patients underwent conversion of MUA/cast to fixation (screws, TSF, IM nail): two conversions were due to fracture displacement during follow-up and one was due to active ingrown toenail infection at presentation; therefore, underwent a staged MUA to improve position before surgery.

Patients were taken to the operating theatre within a median of one day. The length of stay ranged from one to 15 days (median: 2 days). The last follow-up X-ray after the operating theatre visit was at 14 to 564 days (median: 122 days). The number of X-rays taken during follow-up was 1-12 days (median: 4 days). The wide range of X-ray numbers taken post-operatively reflects the heterogeneity of treatment, including a plaster cast only and different types of fixations. For example, a very young patient (e.g., two years old) with simple fracture patterns did not require as many X-rays as an older child (e.g., 16 years old) with delayed union. The principle was to minimize radiation to a child as much as possible by incorporating clinical acumen with fracture patterns. In addition, there was no standard protocol for immobilization after CRIF by flexible nailing. This was entirely surgeon- and case-dependent. The average practice was some form of protected or non-weight bearing initially for two to four weeks. The younger children had less period of immobilization. Children undergoing circular frame fixation were allowed to weight bear as soon as tolerated. In addition, K-wire treated fractures underwent non-weight-bearing mobilization for four to six weeks depending on each case.

Associated conditions included a fall following a recent tibia tubercle osteotomy for patella stabilization; the tibia fracture was continuous with the osteotomy. Other associated injuries were one clavicle fracture, one midfoot dislocation, one metatarsal fracture and one traumatic subarachnoid haemorrhage. The single case of re-fracture in this series was of an 11-year-old boy who presented following a football tackle injury six months after completion of 11-week cast management for a diaphyseal fracture.

Post-operative complications were delayed union, circular frame-related pin site infection and surgical site infection (SSI) following the removal of metalwork (Table 4). The delayed union occurred in the patient with subarachnoid haemorrhage and tibia fracture managed by the circular frame, which ultimately united after seven months of fixation. Infections were successfully treated with antibiotics except for one circular frame pin site infection, which grew enterococcus and needed debridement along with an antibiotic. Another patient treated with the circular frame needed an unplanned return to the operating theatre for re-adjustment. The planned removal of the frame was between one and eight months (median: 10 weeks). The planned removal of all flexible nails (except one) was done between 2 and 15 months (median six months). K-wire, screw and plate planned removal were done after four weeks, five months and six months post-operative period, respectively, for each patient.

**Table 4:** List of post-operative complications

Post-Operative Complication	Frequency	Percent
Delayed union at 7 months	1	1
Infected pin site lysis (circular frame)	3	4
Post-operative infection (surgical site infection after removal of metalwork)	1	1
Total	5 (75)	6

## Discussion

Tibia fractures are common fractures in children and adolescents. In the adolescent age group, this is the third most common long bone fracture [10]. Management options vary according to the patient's age, fracture type, available resources and other factors [7]. Most of the fractures of this age group occur in diaphysis (57%) [11]. Most paediatric tibial fractures, either open or closed, can be managed conservatively by MUA and plaster/casting [12-15]. However, many cases require surgical fixations using various methods [13,16-19]. In our study, no apparent correlation was seen in the distribution of fractures according to age. The diaphyseal fractures occurred in all age groups in our series. Proximal or distal metaphyseal or dia-metaphyseal fractures also happened randomly in all age groups. More than half of the patients were treated with manipulation and casting in the operating theatre, but others needed different fixation techniques to ensure adequate reduction and stability.

Bauer and Lovejoy’s study and Sabatini et al.’s study reported that simple, uncomplicated tibia fractures could be managed conservatively, irrespective of age. The conservative treatment can be achieved by above-knee casting followed by below-knee cast or other means with early return to weight bearing as appropriate to appreciate healing and conservation of normal functional status [20,21]. On the other hand, surgical fixation is indicated when an acceptable position is unachievable with conservative management [22]. Similarly, we also found that conservative treatment was offered to a significant proportion of patients, irrespective of age, whenever possible. However, the incidence of surgical interventions increased significantly with the increase in the age of the patient cohort. Ho et al. reported in a retrospective study that 4% (n=3) of the adolescent patients out of 75 ultimately needed surgical fixation of tibial fractures due to changed position following long leg cast management [23]. The proportion of patients requiring surgery after a period of casting in our series is similar.

Post-operative issues increase the length of hospital stay [24], with significant financial relevance and patients' mental and health well-being issues, especially in children. Patients' length of stay in this series (both casting and surgery) is comparable to the results reported by Weber et al. [9].

Limitations of our study include the retrospective nature and the fact that multiple consultants managed the patients, which influenced treatment choice. However, that did not affect the patient outcome significantly, as we found only a few complications following initial definitive management. Healing time was assessed using clinical and X-ray at the time of outpatient follow-up and therefore was not representative of the actual time of fracture consolidation. We did not find any rotational deformity or significant limb length discrepancy at the final follow-up [25,26].

There were very few open fractures in this study as they were redirected to the regional major trauma centre, which was established around 2012. We did not include patients with tibia fractures, which were managed solely in the outpatient. This may include patients who have had manipulation under sedation in the emergency department. The medical team provides sedation in the emergency department of our institution, but it is not regularly available due to staffing availability.

## Conclusions

Management of tibia fractures in children depends on age, severity and site of involvement. Overall, the complication rates are low. Most young children with tibia fractures can be treated successfully using appropriate casting techniques. Tibia fractures in older children were more commonly managed with surgical fixation. Management efficacy can be improved to decrease the burden on the operating theatre if appropriate sedation, analgesia and monitoring can be established in the emergency department to manage simple fractures of such age group effectively.
